# Sleep in Early Psychosis and Bipolar Disorder: Preliminary Results on Actigraphic and Self‐Reported Markers of Vulnerability

**DOI:** 10.1111/eip.70160

**Published:** 2026-03-10

**Authors:** Valentina Baldini, Francesca Iannucci, Noemi Venezia, Francesco Pasquino, Martina Gnazzo, Diana de Ronchi, Stefano Vandi, Giuseppe Plazzi, Lorenzo Pelizza, Marco Menchetti

**Affiliations:** ^1^ Department of Biomedical and Neuromotor Sciences University of Bologna Bologna Italy; ^2^ Department of Biomedical, Metabolic and Neural Sciences University of Modena and Reggio Emilia Modena Italy; ^3^ IRCCS Istituto Delle Scienze Neurologiche di Bologna (ISNB) Bologna Italy

**Keywords:** actigraphy, bipolar disorder, depression, prodromal symptoms, psychosis, sleep disturbances, suicidal ideation

## Abstract

**Background:**

Sleep disturbances are common in the early stages of psychosis and bipolar disorder and are increasingly regarded as transdiagnostic risk factors for symptom severity and suicidality. However, few studies have simultaneously examined both subjective and objective sleep changes during this early illness phase.

**Methods:**

This cross‐sectional study included patients within 12 months of the onset of psychosis or bipolar disorder and matched healthy controls. Patients completed standardised questionnaires including the Pittsburgh Sleep Quality Index (PSQI), Epworth Sleepiness Scale (ESS), Beck Depression Inventory (BDI), Mood Disorder Questionnaire (MDQ) and Prodromal Questionnaire‐16 (PQ‐16). Actigraphy was recorded for seven consecutive nights in both groups.

**Results:**

Twenty patients (11 with psychosis, 9 with bipolar disorder) and 20 healthy controls were assessed. Most patients reported poor sleep quality (70%), depressive symptoms (60%) and suicidal thoughts (65%). Poor sleep quality was strongly linked to depressive symptoms (*r* = 0.76) and prodromal features (*r* = 0.69). Actigraphy showed reduced total sleep time in patients compared to controls (388 vs. 449 min) and longer sleep latency in bipolar patients than in those with psychosis (161 vs. 80 min).

**Conclusion:**

Sleep disturbances are common during the first year of psychosis and bipolar disorder. Subjective sleep issues are closely linked to depression, prodromal psychotic symptoms and suicidal thoughts, while actigraphy shows disorder‐specific differences. Routine evaluation of both subjective and objective sleep may serve as early indicators of vulnerability and guide intervention strategies in severe mental illness.

## 1 Introduction

Sleep and circadian disturbances are among the most consistent yet underrecognised features of major psychiatric disorders (Wulff et al. 2010). Far from being secondary complaints, changes in sleep timing, continuity and quality are now seen as key aspects of illness expression and potential signs of vulnerability. A growing body of research shows that insomnia, irregular sleep–wake patterns and circadian misalignment are common in both psychotic and bipolar disorders, and these changes often appear early in the course of the illness (Baldini, Pasquino, et al. 2025; Baldini, Gnazzo, Maragno, et al. 2025; Baldini, Gnazzo, Santangelo, et al. 2025; Marchetti et al. 2025). Understanding how sleep interacts with core clinical features in the early stages of psychosis and bipolar disorder may therefore provide important clues for prognosis and early intervention.

In recent‐onset psychosis, most patients report poor sleep quality (Reeve et al. 2019). Subjective complaints of insomnia are often confirmed by objective assessments like actigraphy, which show reduced sleep efficiency (SE), longer sleep onset latency (SOL) and fragmented rest‐activity patterns (Lunsford‐Avery et al. 2015). These changes are not harmless: worse sleep consistently links to more severe positive and negative symptoms, lower cognitive performance, impaired daily functioning and an increased risk of relapse. Furthermore, longitudinal studies indicate that disrupted sleep may not only occur during acute psychotic episodes but also precede them, serving as a potential early warning sign (Afonso et al. 2011).

Sleep disruption is just as common in the early stages of bipolar disorder. Decreased need for sleep and irregular sleep patterns are typical signs of mania, while insomnia and hypersomnia often occur during depressive episodes (Harvey et al. 2005). Even when patients are in a euthymic state, many still report disturbed sleep, which has been linked to a higher risk of relapse. Evidence indicates these changes are not only caused by mood episodes but also serve as predictors of future mood instability (Ritter et al. 2012). In young patients with a recent bipolar diagnosis, abnormal sleep patterns may therefore affect the long‐term course of the illness (Gruber et al. 2009).

Beyond symptom severity, sleep disturbances relate to prodromal phenomena and emotional burden. People at high clinical risk for psychosis often show irregular sleep patterns, and actigraphic studies indicate that these changes predict a greater likelihood of transitioning to psychosis (Lunsford‐Avery et al. 2015; Davies et al. 2017). In early psychosis, fragmented or shortened sleep has been linked to persistent prodromal symptoms, social withdrawal and mood dysregulation (Reeve et al. 2015). Similarly, in bipolar disorder, disrupted sleep may sustain depressive symptoms and slow recovery even outside of acute episodes (Geoffroy et al. 2015). These findings emphasise the broad role of sleep as a mechanism connecting mood and psychotic features from the earliest stages.

A particularly important aspect is the relationship between sleep and suicidality. There is now a strong consensus that insomnia and nightmares are independent predictors of suicidal thoughts, attempts and death by suicide across various populations (Baldini, Gnazzo, Maragno, et al. 2025; Li et al. 2025; Faccini et al. 2025). Importantly, this link remains consistent across different diagnostic categories, age groups, and stages of illness. The underlying mechanisms are still debated, but suggested pathways include impaired emotion regulation, increased impulsivity and changes in stress response systems (Palagini et al. 2025). The first year after the onset of psychosis or bipolar disorder is recognised as a period of increased vulnerability for suicidal behaviour, yet few studies have specifically investigated the role of sleep during this critical stage (Baldini, Gnazzo, Santangelo, et al. 2025; Tubbs and Fernandez 2025). This highlights a notable gap in the research, especially considering the potential for sleep assessment to serve as a simple, non‐invasive and clinically useful tool for suicide prevention.

Consistent with a transdiagnostic perspective, the present study includes both first‐episode psychosis and bipolar disorder to examine shared and disorder‐specific sleep vulnerability markers during the earliest illness phase.

This study aimed to examine sleep disturbances in patients within the first year after developing the first episode of psychotic or bipolar disorder, using both actigraphic and self‐reported measures. Specifically, we sought to explore the links between sleep quality, depressive symptoms, prodromal features and suicidal thoughts. Using a multimodal approach, our goal was to clarify how sleep disturbances influence the clinical presentation of the early stages of the illness and to highlight their potential as targets for early intervention.

## 2 Methods

### 2.1 Study Design and Setting

This was a cross‐sectional observational study conducted at the Bologna Department of Mental Health in Northern Italy, in collaboration with the Narcolepsy Center at the IRCCS Institute of Neurological Sciences of Bologna. The study took place from March 2024 to September 2025, as part of routine outpatient mental healthcare for adults recently diagnosed with psychosis or bipolar disorder.

### 2.2 Participants

Patients were consecutively recruited from community mental healthcare services in Bologna.

Inclusion criteria were: (i) age 18–35 years; (ii) diagnosis of first‐episode psychosis or bipolar disorder, according to the DSM‐5 criteria (APA 2016); (iii) illness episode duration of 24 months or less, consistent with early intervention service criteria, with a specific focus on the first 12 months after illness onset, and (iv) ability to provide informed consent. Exclusion criteria included: (i) intellectual disability, neurodegenerative disease, or major neurological disorder; (ii) comorbid substance use disorder; (iii) inability to complete self‐report questionnaires and (iv) fewer than seven valid nights of actigraphy monitoring.

Sociodemographic and clinical information (such as age, sex, education, employment, living situation, tobacco use, illness duration and current medications) was collected using a structured data sheet. Medication use was not limited, and patients received standard pharmacological treatments as indicated in the international guidelines for the treatment of psychosis and bipolar disorder (NICE 2014) by their psychiatrists.

A group of healthy controls was recruited from university students and young adults in the local community who responded to online postings and word of mouth. Eligibility was verified through a brief clinical interview conducted by a psychiatrist to rule out current or past psychiatric disorders, as well as major neurological or medical conditions affecting sleep. Controls underwent the same actigraphic monitoring protocol as patients. Self‐report questionnaires were not administered to healthy controls, as their role in the study was to provide an objective actigraphic reference for sleep parameters, in line with the pragmatic design of this early‐phase exploratory study and to minimise participant burden.

## 3 Measures

### 3.1 Pittsburgh Sleep Quality Index (PSQI)

The PSQI is a 19‐item questionnaire that measures an individual's sleep quality over the past month. It assesses seven aspects of sleep, including quality, latency, duration, efficiency, sleep disturbances, use of sleep medication and daytime dysfunction. Scores range from 0 to 21, with higher scores indicating poorer sleep quality. A total score above 5 signifies poor sleep quality. This study used the validated Italian version of the PSQI (Curcio et al. 2013).

### 3.2 Epworth Sleepiness Scale (ESS)

The Italian version of the ESS measures subjective sleepiness by asking individuals to rate their likelihood of falling asleep in eight different situations, with possible scores ranging from 0 (would never doze) to 3 (high chance of dozing) on each question (Vignatelli et al. 2003). A total score of ≥ 11/24 indicates pathological subjective excessive daytime sleepiness (Möller et al. 2006).

### 3.3 Beck Depression Inventory (BDI)

Depressive symptomatology was measured using the BDI, a 21‐item self‐report scale with total scores ranging from 0 to 63, where higher scores indicate greater depressive severity. A cutoff of ≥ 14 was used to indicate at least mild depressive symptoms (Beck et al. 1996).

Suicidal ideation was assessed using item 9 of the BDI, which evaluates thoughts of suicide over the past 2 weeks and is scored on a 4‐point scale (0–3). In line with previous research in early‐phase clinical samples, BDI item 9 was analysed both as a continuous variable in correlational analyses and as a binary variable to indicate the presence of suicidal ideation (score ≥ 1 vs. 0) for prevalence estimates and logistic regression models.

### 3.4 Mood Disorder Questionnaire

Lifetime manic and hypomanic symptoms were assessed with the Mood Disorder Questionnaire.

(MDQ) is a widely used 13‐item self‐report screening tool (Hirschfeld et al. 2000). Each item asks for a yes/no answer about experiencing manic or hypomanic symptoms. Besides counting symptoms, the MDQ has two additional questions that check if these experiences occurred at the same time and if they led to moderate to severe functional impairment. While it is not meant to provide a formal diagnosis, the MDQ has been proven to be a reliable tool for identifying bipolar spectrum features in both clinical and community settings.

### 3.5 Prodromal Questionnaire‐16

The Prodromal Questionnaire‐16 (PQ‐16) is a short self‐report screening tool derived from the longer Prodromal Questionnaire, originally developed to identify individuals at increased risk for psychosis (Parabiaghi et al. 2024). It comprises 16 yes/no items assessing positive, negative, and general psychosis‐like experiences (PLEs). Beyond its use in ultra‐high‐risk settings, the PQ‐16 has demonstrated good reliability and validity as a dimensional measure of psychosis‐spectrum experiences in both clinical and research contexts and is widely applied within early psychosis services (Ising et al. 2012).

In the present cross‐sectional study, the PQ‐16 was administered to assess the current presence of PLEs and subthreshold psychotic features, rather than to define a prodromal phase or illness staging. A cut‐off of ≥ 6 on the total symptom score was used to indicate clinically relevant levels of PLEs, in line with previous validation studies (Parabiaghi et al. 2024; Ising et al. 2012).

### 3.6 Actigraphy

Objective sleep–wake activity was recorded using wrist‐worn actigraphs. Participants were instructed to wear the device continuously on the non‐dominant wrist for seven consecutive nights, covering both weekdays and weekends, and to complete a daily sleep diary to assist with data interpretation. Actigraphy data were processed with validated algorithms, providing estimates of total sleep time (TST), SE, SOL, wake after sleep onset (WASO) and circadian rest–activity rhythm. Nights were considered valid if the device was worn for at least 90% of the nocturnal interval; participants with fewer than five valid nights were excluded from the analysis.

### 3.7 Ethics Approval

The study was approved by the Independent Ethics Committee of the “Area Vasta Emilia Centro” (CE‐AVEC) (protocol no. 36330/AUSLBO). All participants provided a signed informed consent form before participating in the study. Each person was free to withdraw their consent at any time.

## 4 Statistical Analysis

Sociodemographic and clinical variables were summarised using means and standard deviations for continuous data, and absolute and relative frequencies for categorical data. Patients were compared based on the type of onset (psychosis vs. bipolar disorder). For continuous data, independent‐sample *t*‐tests or Mann–Whitney *U*‐tests were employed depending on data distribution. For categorical data, chi‐square or Fisher's exact tests were used. Along with *p*‐values, effect sizes were reported using the correlation coefficient (*r*) for non‐parametric comparisons and odds ratios (OR) with 95% confidence intervals (CI) for categorical comparisons.

Associations between self‐reported sleep parameters, depressive symptoms, prodromal features and suicidal ideation were examined using Spearman's rank correlation coefficients.

When examining associations between suicidal ideation (BDI item 9) and depressive symptom severity, the BDI total score was calculated excluding item 9 to avoid item overlap and spurious correlations.

To evaluate clinical significance, questionnaire scores were also categorised based on validated cut‐offs (PSQI > 5, ESS > 10, BDI ≥ 14, PQ‐16 ≥ 6, and endorsement of BDI item 9 for suicidal ideation). Logistic regression models were used to calculate ORs with 95% CIs, comparing the prevalence of above‐threshold values between groups.

Actigraphic variables were averaged across the seven valid recording nights and compared between patients and healthy controls, as well as between psychosis and bipolar subgroups, using *t*‐tests or Mann–Whitney *U*‐tests.

All analyses were two‐tailed, with the significance level set at *p* < 0.05. Effect sizes were reported regardless of significance. Analyses were conducted using JASP (version 12.2; JASP Team, Amsterdam, The Netherlands).

## 5 Results

### 5.1 Sociodemographic and Clinical Characteristics

The final clinical sample had a mean age of 25.00 ± 3.90 years. Slightly more than half were male (55%) (Table 1). Most participants had completed high school (90%), while a small percentage held a university degree (10%). The majority lived with their families (90%), and 30% were employed at the time of assessment. Tobacco use was reported by 15% of the sample.

**TABLE 1:** Sociodemographic and clinical characteristics of the clinical sample.

Variable	Total sample (*n* = 20)	Psychosis (*n* = 11)	Bipolar (*n* = 9)
Gender, *n* (%)			
*Male sex*	11 (55)	7 (64)	4 (44)
Age – M (SD)	25 (3.9)	23 (3.1)	26 (4.6)
Level of education, *n* (%)			
*High school*	18 (90)	11 (100)	6 (67)
*Degree*	2 (10)	0	3 (33)
Employed, *n* (%)			
*Yes*	6 (30)	1 (9)	5 (56)
*No*	14 (70)	10 (91)	4 (44)
Living situation, *n* (%)			
*Alone*	2 (10)	0	2 (22)
*With family*	18 (90)	11 (100)	7 (78)
Tobacco use, *n* (%)	3 (15)	2	1 (11)
Type of onset, *n* (%)			
*Psychotic*	11 (55)	—	—
*Bipolar type I*	7 (35)		
*Bipolar type II*	2 (10)		
Illness duration since onset, *n* (%)			
*< 12 months*	12 (60)	7 (64)	5 (56)
*≥ 12 months*	8 (40)	4 (36)	4 (44)
Pharmacologic treatment, *n* (%)			
First‐generation antipsychotics	1 (5)	1 (5)	0
Second‐generation antipsychotics	11 (55)	7 (64)	4 (44)
Mood stabilisers	9 (45)	1 (5)	8 (89)
Antidepressants	6 (30)	3 (27)	3 (33)
Benzodiazepines	3 (15)	2 (18)	1 (11)

As for clinical features, 11 subjects had a first episode of psychotic onset, while 9 had a first episode of bipolar. Regarding pharmacological treatment, the mean proportions of prescribed medications were as follows: 55% received second‐generation antipsychotics, 45% mood stabilisers, 30% antidepressants and 15% benzodiazepines.

Regarding illness duration, 60% of patients were assessed within the first 12 months after illness onset, while the remaining 40% were evaluated slightly beyond this threshold, reflecting real‐world variability in referral timing within early intervention services.

### 5.2 Group Comparisons

As shown in Table 2, there were no statistically significant differences between patients with first episode psychosis and those with bipolar onset in both sleep and psychopathological self‐reported measures. Also, the prevalence rate of suicidal ideation, assessed through the BDI item 9, was comparable. However, lifetime MDQ manic/hypomanic features tended to be higher in the bipolar group, although this difference did not reach significance (*p* = 0.67). At the same time, PQ‐16 scores were higher in the psychosis group, although not statistically significantly so. Consistently, our results on clinical cut‐offs (Table 3) confirmed these findings. Indeed, none of these proportions differed significantly between the two subgroups.

**TABLE 2:** Comparison of clinical scale scores according to the type of onset.

Variable	Psychosis (*n* = 11)	Bipolar (*n* = 9)	*p*	Effect size (*r*)	95% CI
PSQI, M (SD)	7.0 (3.3)	8.0 (2.7)	0.83	0.16	−0.29–0.53
ESS, M (SD)	7.0 (4.2)	5.0 (2.9)	0.35	−0.26	−0.59–0.21
BDI total, M (SD)	15.0 (10.6)	18.0 (10.4)	0.46	0.14	−0.32–0.51
BDI item 9 positive, *n* (%)	8 (66%)	5 (62%)	0.89	OR = 1.20	0.21–6.80
MDQ, M (SD)	0.2 (0.4)	0.8 (0.3)	0.67	0.63	0.22–0.81
PQ‐16, M (SD)	12.0 (8.8)	7.0 (6.7)	0.76	−0.30	−0.62–0.20

**TABLE 3:** Clinical cut‐offs and prevalence by type of onset.

Variable (cut‐off)	Total sample (*n* = 20)	Psychosis (*n* = 11)	Bipolar (*n* = 9)	OR (95% CI)	*p*
Poor sleep quality (PSQI > 5), *n* (%)	14 (70)	8 (67)	6 (75)	0.67 (0.10–4.35)	0.70
Excessive daytime sleepiness (ESS > 10), *n* (%)	4 (20)	3 (25)	1 (12)	2.33 (0.19–28.0)	0.53
Depression (BDI ≥ 14), *n* (%)	12 (60)	7 (58)	5 (62)	0.86 (0.16–4.73)	0.87
Suicidal ideation (BDI item 9 ≥ 1), *n* (%)	13 (65)	8 (67)	5 (62)	1.20 (0.21–6.80)	0.83
Prodromal symptoms (PQ‐16 ≥ 6), *n* (%)	11 (55)	8 (67)	3 (38)	3.33 (0.55–20.0)	0.21

The correlation matrix among clinical variables is presented in Table 4. Worse sleep quality (i.e., higher PSQI scores) was strongly linked to more severe depressive symptoms (BDI total score; *r* = 0.76, *p* < 0.001) and increased PLEs (PQ‐16 total symptom score; *r* = 0.69, *p* < 0.01). PSQI also had a moderate positive correlation with suicidal ideation, as indicated by the BDI item 9 subscore (*r* = 0.45, *p* < 0.05).

**TABLE 4:** Spearman correlation matrix between clinical variables.

Variable	PSQI	BDI	PQ‐16	ESS	MDQ	BDI item 9
PSQI	1.00	0.76***	0.69**	−0.16	−0.23	0.45*
BDI	0.76***	1.00	0.64**	−0.08	−0.11	0.52*
PQ‐16	0.69**	0.64**	1.00	−0.10	−0.05	0.38
ESS	−0.16	−0.08	−0.10	1.00	0.12	−0.05
MDQ	−0.23	−0.11	−0.05	0.12	1.00	−0.08
BDI item 9	0.45*	0.52*	0.38	−0.05	−0.08	1.00

*Note:* **p < 0.05; **p < 0.01; **p < 0.001.

Depressive symptoms were strongly linked to both suicidal ideation (*r* = 0.52, *p* < 0.05) and prodromal symptoms (*r* = 0.64, *p* < 0.01), further emphasising the connection between affective distress and subthreshold psychotic features. Meanwhile, excessive daytime sleepiness (ESS) and manic/hypomanic features (MDQ) did not show significant links with other clinical variables.

### 5.3 Actigraphic Analyses: Patients Versus Healthy Controls

When compared to healthy controls, patients exhibited a significantly shorter average of TST (388.6 ± 68.4 vs. 449.7 ± 31.8 min; *p* < 0.01). No other actigraphic parameters showed significant differences between these two subgroups (Table 5).

**TABLE 5:** Actigraphic analyses between patients and healthy controls.

Variable	Patients (*n* = 20)	Healthy controls (*n* = 20)	*p*
TST (min)	388.6 (68.4)	449.7 (31.8)	**< 0.01**
SOL (min)	157.7 (110.2)	190.4 (95.6)	0.49
SE (%)	34.1 (26.4)	39.7 (32.6)	0.55
WASO (min)	254.6 (199.4)	229.7 (200.1)	0.69
Wake episodes (*n*)	5.9 (4.4)	4.7 (2.39)	0.31
Sleep episodes (*n*)	5.4 (4.6)	4.2 (3.1)	0.33

*Note:* Bold values indicate statistical significance *p* < 0.05.

Abbreviations: SE = sleep efficiency; SOL = sleep onset latency; TST = total sleep time; WASO = wake after sleep onset.

### 5.4 Actigraphic Analyses: Psychosis Versus Bipolar Onset

Within the clinical sample, patients with bipolar onset showed significantly longer sleep latency compared to those with psychotic onset (160.8 ± 110.5 vs. 80.1 ± 61.4 min; *p* < 0.01). Other actigraphic variables did not differ significantly between groups, although there were trends toward lower SE and a higher number of wake and sleep episodes in the psychosis group (Table 6).

**TABLE 6:** Differences in actigraphic analyses between psychosis and bipolar patients.

Variable	Psychosis (*n* = 11)	Bipolar (*n* = 9)	*p*
TST (min)	392.6 (30.9)	383.6 (89.3)	0.78
SOL (min)	80.1 (61.4)	160.8 (110.5)	**< 0.01**
SE (%)	26.7 (17.8)	43.1 (33.1)	0.17
WASO (min)	276.3 (212.8)	228.4 (190.8)	0.60
Wake episodes (*n*)	7.5 (4.9)	4.1 (3.4)	0.08
Sleep episodes (*n*)	7.1 (4.9)	3.3 (3.2)	0.06

*Note:* Bold values indicate statistical significance *p* < 0.05.

Abbreviations: SE = sleep efficiency; SOL = sleep onset latency; TST = total sleep time; WASO = wake after sleep onset.

## 6 Discussion

This study examined both subjective and actigraphic sleep disturbances in patients during the first year following psychosis or bipolar disorder onset. To our knowledge, it is one of the first to combine both subjective and actigraphic sleep assessments in the initial year of these illnesses. By linking these complementary perspectives to depressive burden, prodromal features, and suicidal ideation, the study highlights sleep disturbances as early, clinically relevant markers of vulnerability. This focus on the earliest stages of illness, along with the dual (subjective and objective) assessment approach, provides a new contribution that expands on previous research involving more chronic samples.

Three main findings emerged. First, self‐reports confirmed that most patients experienced poor sleep quality, high levels of depressive symptoms and suicidal thoughts, with strong links between sleep quality, PLEs and emotional burden. Second, when sleep was objectively measured with actigraphy, patients showed significantly shorter sleep TST compared to healthy controls, while other sleep parameters were similar. Third, within the clinical group, patients with a bipolar onset had notably longer sleep latency than those with a psychotic onset, indicating possible differences in the sleep profile of these early‐stage disorders. Together, these findings highlight that even within the first year of illness, distinct sleep profiles may emerge between psychosis and bipolar disorder. Suicidal ideation was strikingly common (65%) and showed moderate associations with both poor sleep quality (*r* = 0.45, *p* < 0.05) and depressive symptoms (*r* = 0.52, *p* < 0.05). These effect sizes suggest that suicidal thought was not only frequent but also meaningfully linked to both sleep and affective burden. The integration of subjective and objective approaches in this critical phase offers a new perspective compared to prior research, which has mainly been conducted in chronic samples.

Consistent with previous studies, most patients reported poor sleep quality, as indicated by the PSQI, with mean scores significantly above the clinical threshold (Dondé et al. 2022; Bagautdinova et al. 2023; Ong et al. 2020). Rates of clinically relevant depressive symptoms and suicidal ideation were also high, highlighting the emotional burden often seen in the early stages of psychosis and bipolar disorder. Importantly, subjective sleep quality was strongly linked to both depressive symptoms and prodromal features, supporting the idea that disrupted sleep may serve as a transdiagnostic factor that amplifies emotional distress and subthreshold psychotic phenomena. These findings align with earlier research in populations with early psychosis and bipolar, where sleep problems have been associated with increased symptom severity, impaired functioning and a higher risk of relapse (Kaskie et al. 2017; Waters et al. 2013; Cretu et al. 2016). The strong relationship with suicidal thoughts further underscores the clinical importance of routinely assessing sleep as a simple marker of vulnerability.

Actigraphic findings supported these self‐reports by showing objective changes in sleep. Compared to healthy controls, patients exhibited a significant reduction in TST, consistent with previous actigraphy and polysomnography studies indicating shortened or fragmented sleep in early stages of severe mental illness (Lunsford‐Avery et al. 2017; LaGoy et al. 2022; Leseur et al. 2024; Lauderdale et al. 2008). Other actigraphic parameters, such as SE and WASO, did not differ significantly between groups, likely due to high individual variability in sleep among young adults. Within the clinical sample, patients with a bipolar onset had significantly longer SOL than those with a first‐episode psychotic onset. This supports evidence that insomnia is especially common in bipolar disorder, even early on, and may indicate a specific vulnerability to sleep initiation difficulties related to circadian disruption or co‐occurring affective symptoms. Although other actigraphic measures showed only trends toward differences between psychosis and bipolar groups, the latency finding suggests that actigraphy could help identify disorder‐specific sleep patterns from the earliest stages.

This partial agreement supports previous research indicating that self‐reported and actigraphic measures often capture different aspects of sleep disturbance (Jackson et al. 2018). In our study, self‐reports revealed a widespread perception of poor sleep, strongly linked to depressive burden, prodromal features, and suicidality, while actigraphy showed a narrower pattern, with reduced TST and increased SOL in bipolar onset. Rather than a limitation, this difference highlights the complementary nature of subjective and objective assessments. The perceived experience of poor sleep may itself be a meaningful clinical marker of vulnerability, while actigraphy detects dysfunctions that are less obvious to patients. In this way, the discrepancy between subjective and objective measures becomes valuable and warrants clinical attention.

From a clinical perspective, these results emphasise the importance of systematically assessing sleep disturbances in young patients at the onset of psychosis or bipolar disorder. The strong connections between poor subjective sleep quality and depressive symptoms, prodromal features, and suicidal thoughts suggest that simple tools like the PSQI can help identify individuals at higher risk of emotional distress and suicidality. At the same time, actigraphy offers objective markers, such as decreased TST or increased SOL, which may uncover aspects of sleep problems that patients themselves do not easily notice.

Furthermore, combining both self‐report and actigraphic monitoring in early intervention services could enhance risk assessment, guide personalised treatment strategies, and support the early implementation of sleep‐focused therapies in early psychopathologies. Approaches such as CBT‐I, circadian‐based strategies and light therapy should be systematically studied in these early stages, as they may not only improve sleep but also reduce depressive symptoms and the risk of suicide, ultimately leading to better long‐term outcomes and patient's quality of life. Targeting sleep from the very first stages of illness could thus become a critical factor in altering the long‐term course of psychosis and bipolar disorder. Particularly, these data suggest that routine sleep assessments could be a low‐cost, scalable strategy to identify young patients at greatest risk of suicide.

From a translational perspective, the combined use of self‐report tools (like the PSQI) and simple actigraphic monitoring provides a low‐cost, easy‐to‐implement strategy within early intervention services. These tools can offer complementary information on both perceived and objective sleep issues, helping clinicians identify young patients at increased risk for emotional distress and suicidality. In sum, systematically incorporating sleep assessments into routine care could serve as a practical step toward prevention, with the potential to improve functional outcomes and decrease suicide risk in early severe psychopathologies.

Given the small, mixed sample, findings should be considered exploratory and hypothesis‐generating, informing future longitudinal studies designed to disentangle shared versus diagnosis‐specific sleep mechanisms.

This study should be viewed as a preliminary step within an ongoing project. Planned longitudinal analyses will examine trajectories of subjective and objective sleep disturbances and test their predictive value for diagnostic stability, relapse, functional outcomes and suicidal behaviour during the early course of illness.

Despite the small sample size, the consistent relationships observed between subjective sleep quality, depressive symptoms, PLEs and suicidal ideation, as well as the objective markers identified through actigraphy, highlight the potential clinical importance of sleep assessment in the early stages of severe mental illness. Recruitment is ongoing, and future analyses with larger, longitudinal samples will be essential to confirm these findings, evaluate their prognostic value and determine whether early sleep disturbances can predict relapse, functional decline or suicidal behaviour over time.

Several limitations should also be acknowledged. Pharmacological treatment may have influenced both subjective and actigraphic sleep parameters. Antipsychotics, mood stabilisers, antidepressants and benzodiazepines are known to exert heterogeneous effects on sleep architecture, latency and continuity. Given the naturalistic design and limited sample size, medication‐specific effects could not be disentangled from illness‐related sleep disturbances, and the observed patterns likely reflect their combined influence.

The small sample size reduces statistical power and limits generalisability, and the cross‐sectional design prevents drawing conclusions about causality. Suicidality was assessed using a single BDI item, which is a practical choice in early‐stage studies. However, future research should employ validated multi‐item instruments to capture the full spectrum of suicidal phenomena. Healthy controls did not complete self‐report questionnaires; this was an intentional methodological decision aimed at reducing burden in the control group since their primary role was to serve as an actigraphic reference. Future studies should also include subjective sleep quality assessments in controls to enable more direct comparisons. Nonetheless, the observed patterns—shorter TST in patients and longer SOL in those with bipolar onset—align with previous research and are unlikely to be solely due to medication effects. Lastly, the narrow age range of the sample increased homogeneity but limited the applicability of the findings to older populations.

## 7 Conclusion

This study suggests that sleep disturbances are very common during the first year of psychosis and bipolar disorder. Subjective sleep quality is closely linked to depression, prodromal features and suicidal thoughts, while actigraphy shows decreased sleep duration and increased sleep latency. These findings highlight the importance of routine sleep evaluations as an accessible and comprehensive marker of vulnerability in the early stages of severe mental illness.

Future research should expand these findings with larger, long‐term samples to determine if early sleep problems predict relapse, functional outcomes or suicidal behaviour over time. Studies using multiple methods, including actigraphy, polysomnography, circadian markers and ecological momentary assessment, could help clarify the relationship between sleep, mood and psychotic symptoms. Additionally, randomised trials testing sleep‐focused treatments in early psychosis and bipolar disorder are necessary to evaluate their potential in preventing clinical decline and reducing suicide risk.

## Funding

The authors have nothing to report.

## Ethics Statement

This study has received ethics approval from the Independent Ethics Committee of the “Area Vasta Emilia Centro” (CE‐AVEC) (protocol no. 36330/AUSLBO).

## Conflicts of Interest

The authors declare no conflicts of interest.

## Data Availability

The data that support the findings of this study are available from the corresponding author upon reasonable request.
